# Assessing the potential of atomistic molecular dynamics simulations to probe reversible protein-protein recognition and binding

**DOI:** 10.1038/srep10549

**Published:** 2015-05-29

**Authors:** Luciano A. Abriata, Matteo Dal Peraro

**Affiliations:** 1 Laboratory for Biomolecular Modeling, School of Life Sciences, École Polytechnique Fédérale de Lausanne (EPFL) and Swiss Institute of Bioinformatics (SIB), Lausanne, Switzerland

## Abstract

Protein-protein recognition and binding are governed by diffusion, noncovalent forces and conformational flexibility, entangled in a way that only molecular dynamics simulations can dissect at high resolution. Here we exploited ubiquitin’s noncovalent dimerization equilibrium to assess the potential of atomistic simulations to reproduce reversible protein-protein binding, by running submicrosecond simulations of systems with multiple copies of the protein at millimolar concentrations. The simulations essentially fail because they lead to aggregates, yet they reproduce some specificity in the binding interfaces as observed in known covalent and noncovalent ubiquitin dimers. Following similar observations in literature we hint at electrostatics and water descriptions as the main liable force field elements, and propose that their optimization should consider observables relevant to multi-protein systems and unfolded proteins. Within limitations, analysis of binding events suggests salient features of protein-protein recognition and binding, to be retested with improved force fields. Among them, that specific configurations of relative direction and orientation seem to trigger fast binding of two molecules, even over 50 Å distances; that conformational selection can take place within surface-to-surface distances of 10 to 40 Å *i.e.* well before actual intermolecular contact; and that establishment of contacts between molecules further locks their conformations and relative orientations.

Noncovalent interactions between biological macromolecules are the cornerstone of cellular biochemistry^1^,^2^,^3^,^4^,^5^,^6^. Binding of two molecules is governed by the fundamental physics underlying molecular diffusion and intermolecular forces; however, the binding of two biological macromolecules is further complicated by their complex internal dynamics, the poorly understood modulation of dielectric properties inside proteins and at interfaces, and the strong effects from solvent reorganization (due to large changes in solvent-exposed area) and from crowders (arising from the very large solute concentrations inside cells). In a protein-protein binding event all these basic ingredients are entangled, adding up to a complex phenomenon that coordinates the approaching of the interacting molecules, their rotational reorientation, and steps of conformational selection and/or induced fit to lock the complex. The nature, coupling and kinetics of these steps are unclear, because of the lack of experimental methods that can probe individual events at atomic level with sufficient temporal resolution.

In this context, molecular dynamics (MD) simulations stand as a valuable tool with the potential to dissect protein-protein binding at the finest level of detail, especially now given the high accuracy and extensive validation of current molecular force fields (at least for globular, single-protein systems) and the widespread availability of resources for running microsecond simulations of large systems. Several works have so far explored protein-protein binding through simulations; however, those which have accounted for translational and rotational diffusion have employed coarse models and methods that disregard the atomistic details^7^,^8^,^9^, whereas those which have used atomic-level descriptions have simulated the already docked complexes but not the intermolecular approach and reorientation^10^. Other works have combined purely theoretical calculations with short molecular dynamics simulations at specific, fixed configurations of two proteins^11^,^12^. Although undoubtedly insightful on different aspects, those works fail to capture all the important components of intermolecular binding at once, as one could expect from long atomistic MD simulations started from configurations were the proteins are far apart. To the best of our knowledge, no studies have so far employed free atomistic simulations to study reversible protein interactions, and the few available reports focus on more irreversible processes like aggregation, high-affinity dimerization and small-ligand binding^13^,^14^,^15^,^16^,^17^,^18^. In this article, we report our attempts to reproduce through free atomistic MD simulations the noncovalent, reversible dimerization of ubiquitin, which has been recently observed in solution-state NMR experiments at millimolar concentrations^19^.

Ubiquitin is a small, globular, soluble protein that binds covalently to proteins targeting them for degradation^20^. This function requires specific recognition and binding of the different target proteins by different conformational states, highlighting the functional importance of its dynamic features^21^. Standing as a workhorse protein in studies of protein flexibility through NMR experiments and MD simulations, ubiquitin’s internal dynamics have been extensively characterized in different conditions^10^,^11^,^21^,^22^,^23^,^24^,^25^,^26^,^27^,^28^,^29^,^30^,^31^,^32^,^33^,^34^,^35^,^36^. Of most relevance to this work, fully atomistic MD studies have shown that current force fields can accurately describe the ensemble of ubiquitin conformations involved in binding to different targets, with the conformations exchanging in a 50–100 ns timescale *in silico*^10^,^11^.

Under certain circumstances, two or more ubiquitin molecules can bind covalently to each other and to a target protein in a functionally distinct process known as polyubiquitination^20^. This proceeds through condensation of the free primary amines (N-terminus, Lys6, Lys11, Lys48 and Lys63) of one ubiquitin molecule with the terminal carboxylate of another (Fig. 1A)^37^,^38^,^39^,^40^,^41^,^42^,^43^,^44^. These covalent dimers are further stabilized by noncovalent interactions mostly through the surface around Ile44 in the molecule that donates the amino group for the linkage, and around Ile44 or Asp52 in the molecule that donates the terminal carboxylate (Fig. 1A,B, notice that other covalent dimers also exist). But on top of this capacity to dimerize covalently, NMR experiments have shown that ubiquitin dimerizes in solution into a loose, fast-exchanging complex at millimolar concentrations through a surface larger than that observed in covalent dimers^19^ (Fig. 1C). These noncovalent dimers could be functional in regulating polyubiquitination, providing templates for covalent dimerization, or targeting ubiquitin itself for degradation.

In this work we have exploited ubiquitin’s noncovalent dimerization equilibrium to explore the potential of fully atomistic MD simulations as a tool to study reversible protein-protein encounters, recognition and binding. Our main findings are (i) that microsecond simulations at these concentrations ensure effective encounters, but (ii) most of the resulting complexes are not compatible with the NMR data, and (iii) they are too much stabilized. We obtain multimeric aggregates rather than dynamic binding and dissociation of only two protein molecules through the expected noncovalent dimerization interface. On the positive side, though, there is some preference towards interaction through interfaces known to exist in different covalent and noncovalent dimers, indicating that the basic ingredients driving recognition and binding are not exceedingly far from an accurate description. Given similar observations in other recent works, it is clear that improvements are required in the current molecular force fields, as we discuss, especially as bigger multi-molecule systems are simulated for longer timescales. We finally analyze the main aspects of some observed binding events in the context of existing proposals on how long-range recognition and binding proceed, suggesting some salient features of protein-protein recognition and binding to be retested with improved force fields.

## Results

The core of our study consists in ten simulations of systems containing three ubiquitin molecules in the three main fast-exchanging conformations identified in previous works^11^,^34^,^35^, inside cubic boxes of 100 Å edges to achieve concentrations of ~5 mM and placed such that any two atoms from different molecules were not closer than 30 Å in the initial configuration. Our choice of including three protein molecules stems from the idea that they should, according to the reported dimerization equilibrium and under sufficiently long and accurate sampling, lead to specific binding of two molecules with the third one remaining alone, possibly exchanging later with one of the first two after dissociation of the dimer. Of the ten replicas, five were set up in pure water and five in 100 mM Na^+^ and Cl^−^ ions to match the ionic strength in the NMR experiments^19^. The neutral charge of the ubiquitin molecules also matches the conditions of the NMR experiments, carried out at pH 7 being the isoelectric point of ubiquitin 6.8. After minimization of these systems and equilibration to 300 K, ~600–900 ns of simulation were produced for each. Proteins and explicit water were described with AMBER99SB^45^ and TIP3P^46^ parameters. In one additional system of three ubiquitin molecules in 100 mM NaCl we used the more recent AMBER99SB-ILDN^47^ parameters for the proteins, obtaining the same results that we obtained with AMBER99SB thus ensuring that the improvements entailed by the ILDN correction do not affect the performance of the core force field in capturing reversible dimerization.

### Encounters and interactions readily take place in submicrosecond simulations at over 5 mM ubiquitin concentration

**Figure 2:**
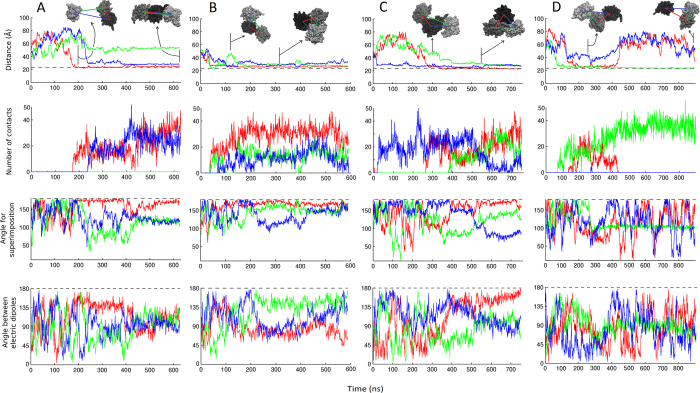
Inter-protein distances, angles and number of contacts in selected MD simulations of three ubiquitin molecules at over 5 mM concentration. Simulations in panels **A** and **B** are in water, those in panels **C** and **D** are in 100 mM NaCl concentration. In the plots reporting distances, angles and number of contacts, red, green and blue correspond to molecule pairs 1-2, 1-3 and 2-3, respectively. Dashed lines correspond to the gyration diameter of ubiquitin (23.4 Å) in the distance plots and to 180° in the angle plots. Relevant structural snapshots of dimers and trimers as extracted from the trajectories are shown on top.

Figure 2 shows the time evolution of protein-protein distances, angles and contacts for the three possible pairs of molecules in four selected simulations (Figure S1 shows plots for all 10 simulations). The reported distances are those between centers of mass, expected to be minimal and close to the gyration diameter (23.4 Å) in a compact dimer. Protein-protein angles correspond to the total angle required to fit one protein on the other after having centered them on their centers of mass, thus reporting on their relative orientations (the maximum possible value of 180° corresponds to a perfect dimer of C_2_ symmetry). Protein-protein contacts were defined as the number of heavy atoms of each protein being within 4 Å of heavy atoms of the other. Also the angles between the dipolar moments of molecule pairs are shown.

In the first 50 ns of our ten simulations containing three protein molecules, when they are still far from each other, their diffusion coefficients range from 2 to 5·10^−11^ m^2^/s which corresponds to a Brownian sampling of 110–173 Å per microsecond. Considering these estimates and that ubiquitin’s gyration diameter is roughly one fourth of the box edge length, it seems in principle likely to observe encounters between pairs of ubiquitin molecules in a submicrosecond timescale. In turn, given the reported dimerization equilibrium it is also possible that some of these interactions could be favorable and result in the formation and further dissociation of dimeric species at similar rates. Encounters between ubiquitin molecules to form dimers are indeed readily observed in all the ten simulations (Fig. 2 and S1); moreover, most of the formed dimers grow into trimers during the simulation times. The fastest binding event occurs in around 20 ns whereas most others take around 100–300 ns with no apparent effect of the presence of NaCl (which does not aggregate as found for other salts in long simulations^48^). Before any encounter between two molecules, the protein-protein angle and the angle between dipolar moments fluctuate amply, indicating nearly free tumbling and no strong interaction. After binding, the angles quickly adopt a defined value, sometimes after a slight adjustment during which the molecules “roll” on each other. All the obtained complexes entail large numbers of contacts; but as we develop below, most sets of contacts are inconsistent with the NMR data about noncovalent dimerization except for the interaction between molecules 1 and 2 in the simulation presented in Fig. 2A (compare Figure S2A against S2B–D).

### All simulations ultimately result in aggregates, indicating that some forces are unbalanced leading to overestimated protein-protein interactions

The establishment of many long-lasting interactions and the formation of trimers in the simulations disclose a strong tendency to aggregation, conflicting with the equilibrium experimentally observed at millimolar concentrations. Binding events observed in the simulations take place within tens to hundreds of nanoseconds, and dissociations should take place at a similar rate but they rarely occur. Although it is possible that they simply lie on a slightly slower timescale, the observation of compact aggregates with more than two molecules and the binding of molecules through interfaces different than that determined by NMR suggest *per se* that the interactions are too strong in the simulations. We review in the discussion section that this shortcoming is consistent with several observations in other recent works, and elaborate on its possible sources.

A brief summary of aggregation scenarios follows. In some replicas like that in Fig. 2B, the three molecules happen to rapidly establish contacts with each other within 50–100 ns, directly forming compact trimeric assemblies that last for the rest of the simulations. In most other replicas the process takes longer and involves initial formation of a dimer, but the third protein inevitably ends up joining to form either directly a compact trimeric cluster where each molecule interacts with the other two, or a (nearly) linear arrangement of three molecules where they interact only pairwise and which subsequently rearranges into a trimeric cluster. Notice that the opposite (*i.e.* conversion of a compact trimer into a linear trimer) is never observed, consistent with a tendency to compaction and overestimated packing. Likewise, dissociations are barely observed; actually only one full dissociation event occurred (Fig. 2C).

The tendency to aggregate irreversibly was also clear in simulations carried out with different numbers of protein molecules (2 to 30 molecules, ranging from 3 to 50 mM concentration), all of which resulted in clustering and large compaction (some states are shown in Figure S4). Finally, to further make the point that protein-protein interactions are too much stabilized, we note that exploratory metadynamics simulations increasing the distance between the centers of mass of proteins engaged in different dimers indicate that 5 to 20 kcal/mol are required to dissociate them (not shown), which would very roughly correspond to dissociation timescales in the order of 10^−2^ to 10^9^ seconds assuming a preexponential factor of 1 μs^−1^.

### Despite the tendency to aggregation, the simulations do favor binding through interfaces that exist in noncovalent dimers and in covalently linked ubiquitin

**Figure 3:**
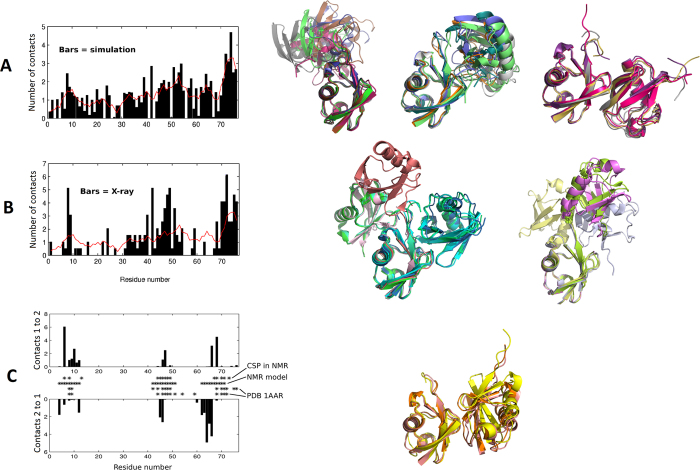
Dimers extracted from the MD simulations, compared against structures of covalent dimers and NMR data about noncovalent dimers. (**A**-**B**) Average number of contacts observed per residue in the ten simulations of three ubiquitin molecules (A, black bars) and in X-ray structures of covalent ubiquitin dimers (B, black bars). In both plots, the red trace is the running residue-average of the MD data. Representative structures are shown on the right for complexes formed in the MD simulations (authentic dimers and dimers extracted from trimers) and for X-ray structures, aligned so as to highlight similarities and differences. Panel **C** compares the trajectory-averaged residue contacts observed in the NMR-compatible dimer formed in the trajectory in Fig. 2A against NMR data about ubiquitin dimerization Here, CSP stands for Chemical Shift Perturbation, NMR model points at contacts described by Liu *et al*. for their NMR model, and PDB 1AAR points at contacts observed in that PDB structure for covalent diubiquitin.

The preceding description of the observed events as irreversible aggregation reveals a clear overestimation of binding energies and suggests a lack of specificity in the interactions. However, visual inspection of the trajectories and of individual contacts plots like those of Figure S2 suggest some degree of preferential binding on certain regions of the protein. This is quantified in Fig. 3A as the number of times each residue is involved in a contact, averaged across the ten simulations. The offset in this plot indicates that most exposed residues are involved in interactions at some point; however, there are local maxima of preferential interaction at around residues 8–9, 20, 24, 39–42, 47–54, 62–66 and 71–76. Interactions observed between ubiquitin molecules in X-rays structures of covalent dimers are localized to residues 8-9, 24, 40, 42, 46–51 and 70–76 (Fig. 3B), overlapping reasonably well with the maxima observed in the MD profile. Accordingly, the most representative dimers from the simulations (Fig. 3A on the right, either free or extracted from trimers) are mediated primarily through the same interfaces involved in covalent dimerization (Fig. 3B on the right). Notice that the dimers are flexible in MD in contrast to nearly frozen dynamics in protein crystals; such flexibility surely contributes to blur out the maxima in the MD data and thus aggravate the match to the X-ray profile.

The specific segment spanning residues 62 to 66 establishes many inter-protein contacts in the MD simulations but very few in the X-ray structures of covalent dimers (Fig. 3A,B). This segment is involved in noncovalent dimerization of ubiquitin as revealed by NMR (extending up to residue 71), together with segments 4–12 and 42–51 (asterisks in Fig. 3C). Notice, though, that the combination of three segments consistent with NMR data was observed to form stable dimers only once in all our simulations (that in Fig. 2A). Many other interactions took place through segment 62–66, but were accompanied by other interactions that do not match all the NMR-derived contacts at once.

### Analysis of the noncovalent dimerization event compatible with the NMR data

**Figure 4:**
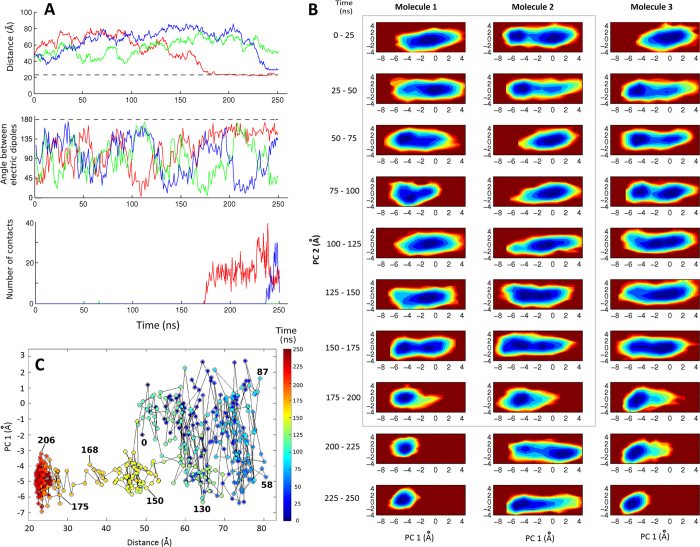
Dissecting the formation of an NMR-compatible dimer. (**A**) Inter-protein distances, angles and contacts during the first 250 ns of the simulation presented in Fig. 2A. Trace colors are as in Fig. 2, the red one corresponding to the binding of molecules 1 and 2. (**B**) Conformational space covered by the three protein molecules during 25 ns intervals, provided by the projection of the trajectory on the first two principal components describing ubiquitin variability across X-ray structures. Molecules 1 and 2, inside the gray box, are the two that bind to give the NMR-compatible dimer (*i.e.* red traces in panel A). (**C**) Plot of the conformational state of molecule 1 as measured by PC1, against the distance of its center of mass to that of molecule 2 (plotted every 0.5 ns, some relevant time points are given).

We now describe the formation of the above-mentioned dimer consistent with NMR data about noncovalent dimerization (Fig. 3C), which occurs in an event during which the third ubiquitin molecule remains always more than 40 Å away from the two binding proteins (first 250 ns in Fig. 2A). Figure 4A zooms on Fig. 2A to show the distance, dipole angle and number of contacts between pairs of protein molecules in the first 250 ns of the analyzed trajectory. Figure 4B displays projections of the trajectories of the three proteins over 25 ns windows on a PCA frame that gathers in 2 principal components over 55% of the structural variability observed in X-ray structures of ubiquitin (projections over 50 ns windows for the full trajectory are shown in Figure S3). Figure 4C displays the position along PC1 (which covers 42.6% of the variability) for molecule 1 as a function of its distance to molecule 2, color-coded by simulation time.

As shown in many works, an isolated ubiquitin molecule simulated in water explores in 50 ns most of the defined conformational space^10^,^11^,^34^,^35^. This holds for all the molecules in the initial part of all our simulations, when they are still far from each other (four examples in Figure S3). In the event leading to the NMR-compatible dimer, the three proteins freely tumble and explore the conformational space for 150 ns during which they never get closer than 35 Å, and in particular molecules 1 and 2 never get closer than 50 Å (red trace in Fig. 4A and from blue to green to yellow in Fig. 4C). Between 150 and 175 ns, molecules 1 and 2 start to move towards each other getting closer at a relative speed of around 1 Å/ns. The first contact takes place at almost 175 ns, when the centers of mass are still not at their minimal possible distance and the angle between the two proteins is still far from its final value of *ca*. 180°. Initial contacts take place through residues Lys6, Thr9, Gly10, Lys11 and Thr12 of one monomer and residues Phe45, Ala46, Gln62 and Ser65 of the other. It takes additional ~10 ns for the centers of mass to reach their final distance close to twice the gyration radius, during which the molecules get their final orientation and establish more contacts. Naturally, binding of the two proteins results in exclusion of water molecules from the space located between the approaching surfaces, especially in the last stages of the binding event (Figure S5).

In the next paragraphs we explore in more detail how recognition and binding occur. Between 160 and 175 ns, the distance between the two proteins decays roughly exponentially, indicating there seems to be a specific configuration that triggers binding at 160 ns. In that moment, the proteins are separated by ~50 Å with their dipolar moments forming an angle of ~135°. At such long distances only the electrostatic interactions are significant, and thus they must be defining the binding configuration. In this particular case, we estimate an electrostatic attractive force of ~0.1 – 0.5 pN acting on each protein. The first part of the decay in the protein-protein distance barely follows the angle formed by the dipole moments up to 165 ns, possibly because the electrostatic force is still weak to compete with diffusion and other forces. But from that moment, where the dipoles form an angle of 70° and are separated by 35 Å implying an electrostatic attractive force of ~0.5 - 1 pN, the rest of the binding event is uniform with the molecular reorientations following the decrease in protein-protein distance until the dipolar moments get nearly antiparallel (minimizing the potential energy) and the two proteins establish full contact.

Regarding the conformational changes that take place during binding, notice that by 150 ns and at a distance close to 50 Å, before any contact, the two molecules experience a conformational shift towards low values of PC1 (Fig. 4B in the 150–175 ns range for molecule 1 and the 125–150 ns for molecule 2; more explicit for molecule 1 in the region of yellow dots in Fig. 4C). This conformation preexists in the ensemble (when the molecules are far from each other or as observed in simulations of single ubiquitin molecules in water) and is thus selected over a long distance. During the next 50 ns, in which full binding completes, the two proteins increasingly lock in the leftmost basin, *i.e.* binding shifts from conformational selection to induced fit. (Unfortunately, binding of the third molecule by 225 – 250 ns perturbs the conformational fluctuations of molecule 2, hence conformational selection for this molecule is clear between 175 and 200 ns but subsequently lost).

Overall, these observations suggest that recognition and conformational selection can take place over a long distance well before actual contacts are established, induced by long-range electrostatics, and that conformational selection can shift into induced fit as the complex is fastened and as the two dipolar moments acquire the antiparallel orientation that minimizes energy. As a note of caution, we point out that the exact extent of conformational selection and induced fit, as well as the distances over which conformational selection is induced, may be affected by the unbalanced forces that lead to aggregation.

## Discussion

Molecular dynamics simulations continuously profit from the increase in computational power, allowing for investigations on more relevant size- and time-scales. It is not granted, though, that force field development accompanies hardware speedup, hence it is important to continuously benchmark and tune our simulation methods. Of utmost relevance, whereas atomistic simulations are widely employed to understand either single proteins or already formed, stable complexes, and while single-protein simulations are widely used to test and optimize MD methods, not many studies have addressed systems containing multiple copies of a protein investigated at atomic level over microsecond time scales. The studies presented here are thus pioneering in this regard.

There are at least three reasons why our study system represents a very challenging benchmark. First, the noncovalent dimer has low affinity and is not defined by only one clear interface but rather by a large interface where many relative orientations are possible as shown by the NMR experiments. Second, this interface competes in the simulation with other interfaces that are important upon covalent dimerization. Third, ubiquitin has no charge at pH 7 (at which the NMR experiments were carried out and as in the simulated conditions), preventing any net long-range repulsion that could help prevent aggregation. These reasons can be summarized in the interaction being weak, fast-exchanging and of low specificity.

### Needs for force field improvement

A few previous works studied oligomerization events at atomistic level but on systems that are expected to bind irreversibly in the time scales of the simulations, such as during folding of a dimer, aggregation to form amyloid fibers, ligand binding, etc.^13^,^14^,^15^,^16^,^17^,^18^ In those works, the lack of dissociation events did not imply problems, whereas for the reasons mentioned above our simulations allowed us to pinpoint this problem. Moreover, the available data about covalent and noncovalent dimerization allowed us to evaluate the different interfaces.

The tendency to aggregation has shown up in other recent works, although it was not always discussed. And apparently, it is a problem that affects all of the most popular force fields. A very recent work showed that current versions of popular force fields (including AMBER99SB-ILDN, CHARM22-CMAP, GROMOS 45a3 and 54a7, and OPLS-AAL) are not accurate enough to study proteins in crowded environments because of excessive tendency to interaction^49^. Another recent work employing multi-microsecond atomistic simulations with CHARMM36 reported that free diffusion of two protein molecules led to complex formation in all of 14 independent attempts carried out, but that only one of the obtained complexes satisfied known experimental data^50^. Other atomistic simulations of concentrated protein solutions parameterized with the CHARMM27 force field also produced extensive contacts among proteins^51^, if not aggregation, although they were not nor discussed. And although not described under Results, we also observed aggregation of three ubiquitin molecules within a few hundreds of nanoseconds in one simulation with the AMBER99SB-ILDN force field.

In all the commented cases, and as observed in this work, compaction tends to be maximized. This is similar to the observation with recent versions of the AMBER, CHARMM and OPLS force fields that unfolded proteins tend to be much too compact in simulations relative to what experiments show^52^,^53^, and relates to the fact that small force fields tweaks affect the resulting compaction^54^. All these clues point at an overestimated hydrophobic collapse, and suggest that among the multiple force field elements to blame, defects in the water models and in the treatment of electrostatics are especially important. Recent articles have indeed highlighted the importance of incorporating multipole electrostatics, variable dielectrics and polarizability in atomistic force fields, especially to better reproduce hydrogen bonds, water effects, aromatic stacking, ion-aromatic and other noncovalent interactions^55^,^56^. In particular, the observed tendency to compaction suggests a very important contribution from an inaccurate water description, which seems to lack the strength required to quickly dissociate bad complexes or even prevent their formation. Although there are more advanced water models than the TIP3P model used here, they are still much less validated and used than it, possibly because the most confident force fields for proteins have been tuned themselves with TIP3P water as the solvent.

The gradual introduction of new water models and methods that account for protein and solvent polarizability^53^,^57^,^58^,^59^,^60^,^61^,^62^,^63^,^64^,^65^,^66^ is promising regarding the problems here discussed. As pointed out by Petrov and Zagrovic^49^, this is of utmost importance as the atomistic treatment of crowded environments and large multicomponent systems is increasingly pursued^51^,^67^,^68^,^69^. We note finally that force field parameterization and testing has recently progressed by comparing experimental observables, mainly from NMR, to their predictions from simulations, but the commonly used observables are mostly applicable to small monomeric globular proteins. We propose here that other observables applicable to multiprotein systems (and possibly others applicable to unfolded peptides, which are also poorly described by simulations) should be considered for tuning force fields.

### Diffusion, reorientation and internal dynamics upon recognition and noncovalent interaction.

Recent computational works have studied how proteins recognize each other over different distances and configurations and how conformational dynamics respond to binding, through atomistic simulations started from preset arrangements of ubiquitin and interacting partners but disregarding free diffusion^10^,^11^,^70^. Other works have instead studied binding by allowing free diffusion but with coarse molecular descriptions that could not reflect atomic details nor conformational dynamics^7^,^8^,^9^. To the best of our knowledge, ours is the first study based on unbiased atomistic simulations started from well-separated protein molecules, and as such, it is worth comparing our observations and conclusions to those of other works. Because of the identified shortcomings, though, we have focused only on gross effects.

The works cited above all agree in that protein-protein recognition and binding proceeds through a superposition of conformational selection and induced fit, starting at least at surface-to-surface distances of 10 to 20 Å (which would correspond to a distance between centers of mass of roughly 30 to 40 Å for a ubiquitin dimer). Long and Brüschweiler further proposed that recognition takes place early on in the binding process when long-range electrostatic forces favor specific orientations of the two intervening proteins, before any conformational shifts, at distances of 20 – 40 Å^12^. In our simulations, the dimerization event compatible with NMR data also supports a superposition of conformational selection dominating at 40–50 Å, and induced fit dominating at short distances, although their exact balance can be perturbed as a consequence of the same inaccuracies that lead to aggregation (a caveat not discussed in other works but also applicable to them). We propose that conformational selection slowly blends and shifts into induced fit as the proteins approach, their dipolar moments adopt an antiparallel orientation, and intermolecular contacts are established. But in principle, we cannot fully support the idea that full directional selection must take place beforehand because the two proteins are seen to reorient after having made contact, although the fast exponential decay observed for the protein-protein distance suggests that binding is in fact seeded by special configurations, presumably specific combinations of relative directions and orientations that build up the required long-range electrostatic attraction.

The existence of certain configurations that trigger rapid binding also holds for the events that do not lead to an NMR-compatible dimer, where either the forces that drive binding are too strong and/or the opposing forces are too weak, the latter likely attributable to the water description as discussed above. As a mere example, binding of a third protein molecule to the NMR-compatible noncovalent dimer (Figs. 2A and 4) proceeds very rapidly between 220 and 250 ns, starting from a configuration in which the two proteins from the dimer are 55 – 60 Å away from the third (joining) protein. In this case, the joining molecule experiences conformational restriction long before binding, even at distances longer than 50 Å, and gets locked in a conformational basin after binding which in turn disturbs the conformation of the dimer-engaged molecule to which it binds (Fig. 4). Similar fast decays of the inter-protein distances, accompanied by long-range conformational perturbations, are observed throughout the ten simulations when two proteins bind. And in most cases, conformational selection before binding is followed by induced fit as contacts are established and as the electric dipoles reorient.

Regarding the nature of the configuration that seeds binding, we notice that proximity between proteins is not enough to trigger it, possibly because the direction, angles and/or conformational states in that moment do not produce sufficient attractive interactions. Indeed, many pairs of proteins come closer than 50 Å many times over several tenths to a few hundred nanoseconds (for example molecules 1 and 3 in Fig. 2A or Fig. 4A) yet they do not engage in binding. Thus, it is likely that there is a mixture of directional, orientational, and conformational selection that defines routes for productive encounters, although the forces themselves must be mainly electrostatic for they are the only operative ones at the involved distances.

We finally comment on dissociation events. In a few cases we observe that transient, unproductive interactions mediated by few contacts are established but quickly terminated. In an opposite case, a dimer mediated by many contacts but incompatible with noncovalent dimerization exists for around 150 – 200 ns and then dissociates (by 400–450 ns, Fig. 2D). Dissociations from very short-lived complexes seem to correspond to a “bounce” along the diffusion path, while full dissociation of the long-lived complex seems to respond to a specific repulsive force (in this case arising from the parallel orientation of the electric dipoles right before 400 ns).

In summary, despite the limitations of the simulations, the derived conclusions (i) go in hand with the growing picture of initial conformational selection blending into induced fit during protein-protein binding, (ii) show that conformational selection can be induced across long distances, and (iii) they suggest that predefined directions and orientations exist that seed binding and dissociation, taking place in a few tens of nanoseconds once setup. We note here that the large dipolar moment of proteins^71^, even those of no net charge like ubiquitin, would be very important to modulate binding through relatively large distances. We cannot infer further solid conclusions due to the shortcomings of current simulations, as discussed, but we are confident that slightly improved simulation methods will allow in the near future a better description of the binding equilibria including thermodynamics, kinetics and structural details at atomistic level.

## Methods

Human ubiquitin was taken from conformations observed in our previous work^35^. Systems (of around 130,000 atoms) were built with Packmol^72^ and Ambertools^73^. Simulations were run with the NAMD engine^74^ using AMBER99SB^45^ (or AMBER99SB-ILDN^47^ in one case) and TIP3P parameters^46^. Standard sodium and chloride parameters from the AMBER force fields were used; we checked they did not aggregate nor cluster as found for other salts in long simulations^48^. Each system was minimized and copies were independently equilibrated to 1 atm and 300 K with constraints on the Cα atoms, and then simulated for over 600 ns each. Particle-Mesh Ewald summations^75^ were used to treat electrostatics, with a grid size of 1 Å. The cut-off for nonbonded interactions was set at the conservative^76^ value of 12 Å with a switching function reaching 13.5 Å. The integration time step was 1 fs for equilibrations and 2 fs for production, using the RATTLE algorithm on all bonds to hydrogen atoms. Pressure and temperature were controlled with a Nosé-Hoover Langevin piston during production. Upon minimization and equilibration the simulation boxes shrank raising the ubiquitin concentration by around 20% in all simulations. This implies an expected 2 mM concentration for dimeric ubiquitin considering the dissociation constant determined by NMR, which should favor the observation of dimerization events or at least encounters. Metadynamics simulations^77^ were run with the same parameters in NAMD, adding energy hills of 0.1 kcal/mol at a width of 0.1 Å every 100 ps to drive exploration of the collective variable which was the distance between the centers of mass of both proteins.

Projection of the trajectories on a 2D plane was done as described in our previous works following previous methods^11^,^21^,^34^,^35^. Briefly, a reference frame was built through principal components analysis of the covariance matrix of Cα positions for residues 2–70 of ubiquitin molecules in 72 high-resolution X-ray structures of the protein bound to different targets. The two first principal components account for 42.6% and 12.5% of the structural variability. Snapshots of individual trajectories were projected on that PCA frame and the resulting set of 2D points was used to compute logarithmic probability distributions on binned grids according to *−k*_*B*_*T ln(N*_*i*_*/N*_*0*_), where *N*_*0*_ corresponds to the most populated bin.

Electrostatic interactions between ubiquitin dipolar moments were estimated from the angles *θ* and distances *r* between dipoles as *|*μ*|*^*2*^*(1−3cos*^*2*^*θ)N*_*A*_*/(4πε*_*0*_*Dr*^*3*^) where *ε*_*0*_ is the permittivity of vacuum, *D = 78* accounts for water dielectric constant, N_A_ is Avogadro’s number, and considering that ubiquitin’s dipolar moment μ fluctuates between 200 and 300 D as retrieved from the MD simulations. The electrostatic forces were computed as the first derivative relative to the distance, scaled by *N*_*A*_ to get the force per molecule.

## Additional Information

**How to cite this article**: Abriata, L. A. and Dal Peraro, M. Assessing the potential of atomistic molecular dynamics simulations to probe reversible protein-protein recognition and binding. *Sci. Rep.* **5**, 10549; doi: 10.1038/srep10549 (2015).

## Supplementary Material

Supplementary Information

## Figures and Tables

**Figure 1:**
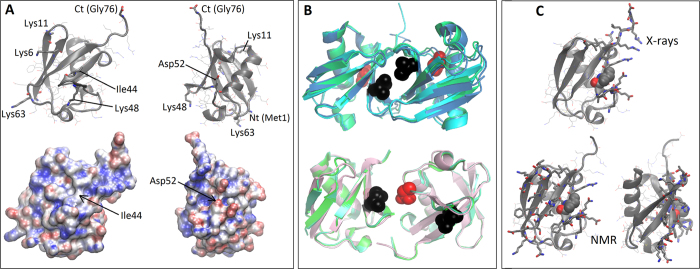
Covalent and noncovalent dimerization of ubiquitin. (**A**) Ubiquitin in cartoon and electrostatic surface representations with relevant residues rendered as sticks. (Two views differencing a 90° rotation are reported). (**B**) Examples of covalent linkages commonly observed in X-ray structures of covalent ubiquitin dimers, with Ile44 and Asp52 rendered as black and red spheres, respectively. Top, PDB IDs 1AAR, 3M3J and 2O6V where the interaction is mediated by the surface around Ile44 on both proteins; bottom, PDB IDs 1TBE, 3NS8 and 3AUL where the interaction is mediated by surfaces around Ile44 in the amino donor and around Asp52 in the carboxylate donor. (**C**) Residues (sticks) involved in contacts in crystallographic covalent dimers (top) or in noncovalent dimerization (bottom) as determined by NMR experiments (Ile44 is rendered as spheres for reference).
