# Supplementary material for: Assessing the potential of atomistic molecular dynamics simulations to probe reversible protein-protein recognition and binding
Source: Sci Rep. 2015 May 29;5:10549. doi: 10.1038/srep10549 (PMC4448524; doi:10.1038/srep10549)
Supplement: Supplementary Information [file srep10549-s1.pdf]

Supporting information for

Assessing the potential of atomistic molecular dynamics simulations to probe reversible protein-protein recognition and binding

Luciano A. Abriata and Matteo Dal Peraro

Laboratory for Biomolecular Modeling, School of Life Sciences, École Polytechnique Fédérale de Lausanne and Swiss Institute of Bioinformatics, Switzerland

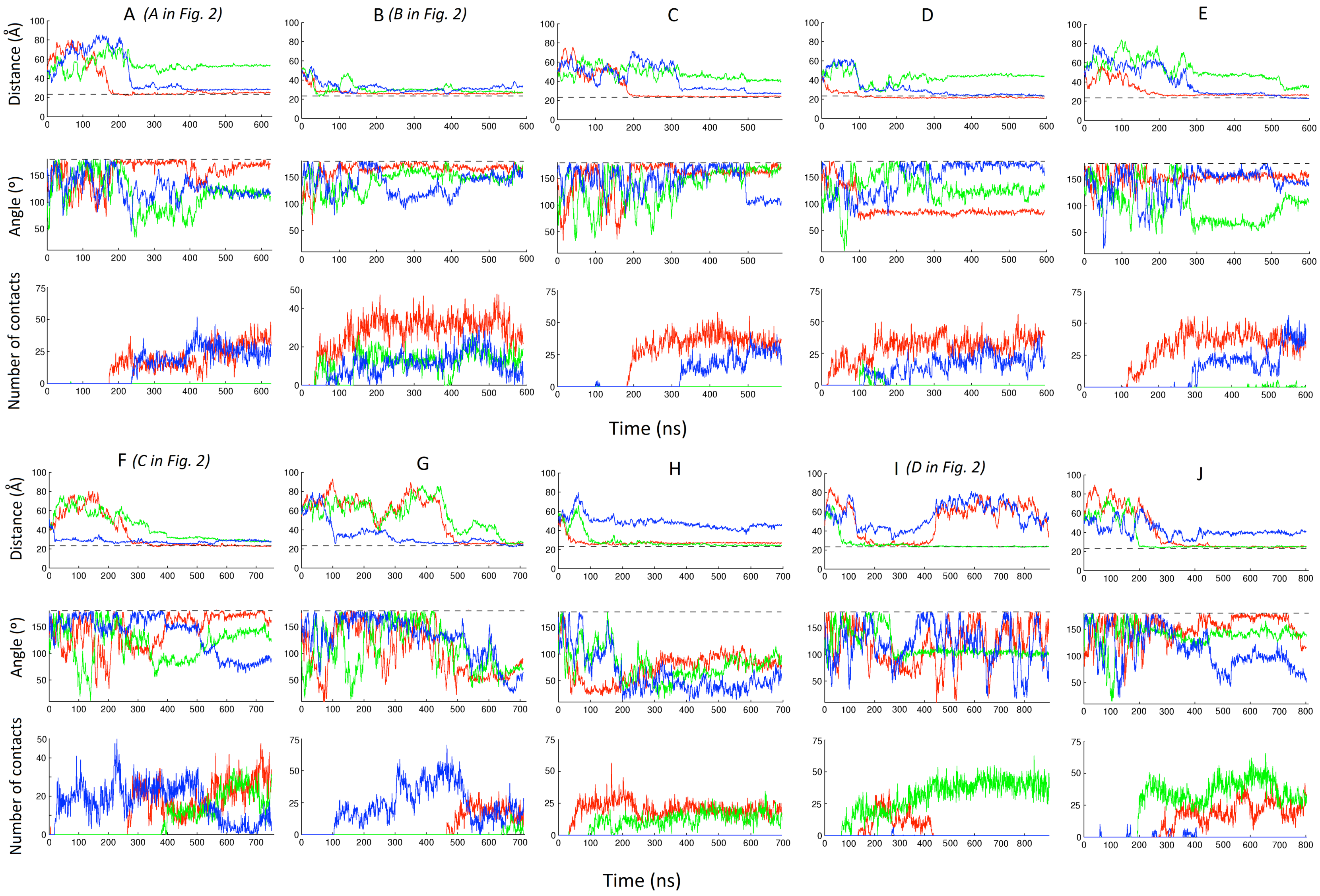

Figure S1. Protein-protein distances, angles and number of contacts in the ten simulations of three protein molecules at 5 mM concentration. Results from ten simulations of systems containing three proteins in boxes of size around 100^3 $Å^3$. Simulations in panels A-E are in pure TIP3P water, those in panels F-J contain also 100 mM sodium and chloride ions. Traces in red, green and blue correspond to molecule pairs 1-2, 1-3 and 2-3, respectively. The dashed lines in the distance plots correspond to the gyration diameter of ubiquitin (23.4 Å) and those in the angle plots

correspond to 180º. The short protein-protein distances, fixed angles and high number of contacts evidence the tendency to aggregation. Figures 2, S2 and S3 show data about contacts and conformational space exploration in four selected simulations. Figure S4 shows snapshots of some aggregates.

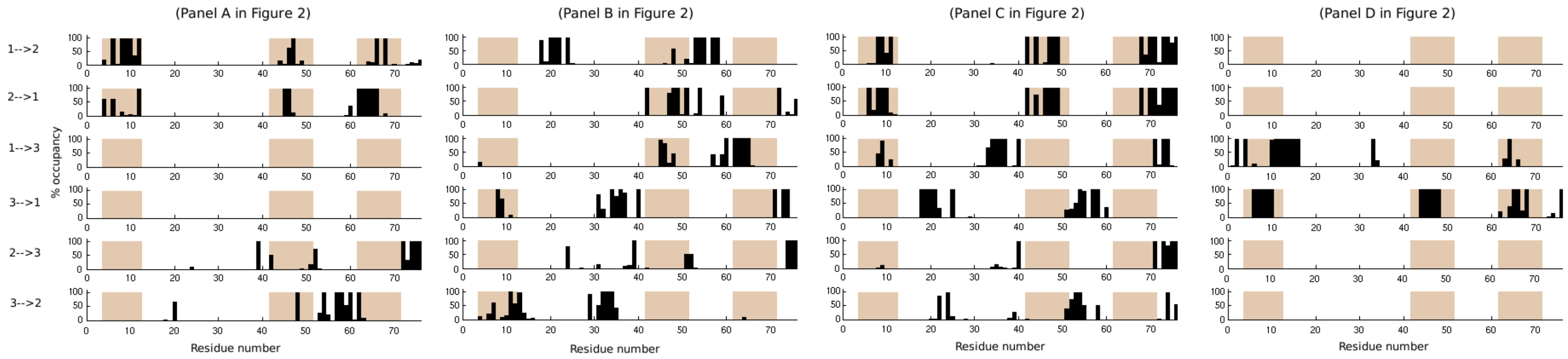

Figure S2. Residues involved in contacts between pairs of ubiquitin molecules, in the four simulations shown in Figure 1 of the main text, quantified as the fraction of time in which the residue was making a contact in the last 200 ns of simulation (the plots focus on the last 200 ns so as to highlight what residues make interactions in the aggregates). On the left, from top to bottom, 1-->2 stands for residues of molecule 1 contacting residues of molecule 2; similarly, 2-->1 stands for residues of molecule 2 contacting residues of molecule 1, and so on. The shaded boxes encompass the three segments of residues that make contact in the noncovalent dimer according to NMR data. Some protein pairs establish contacts very consistent with the NMR data about noncovalent dimerization (like in panel A between 1 and 2) while others are partially consistent (like contacts between 1 and 2 in panel C or between 1 and 3 in panel D) and most are totally inconsistent.

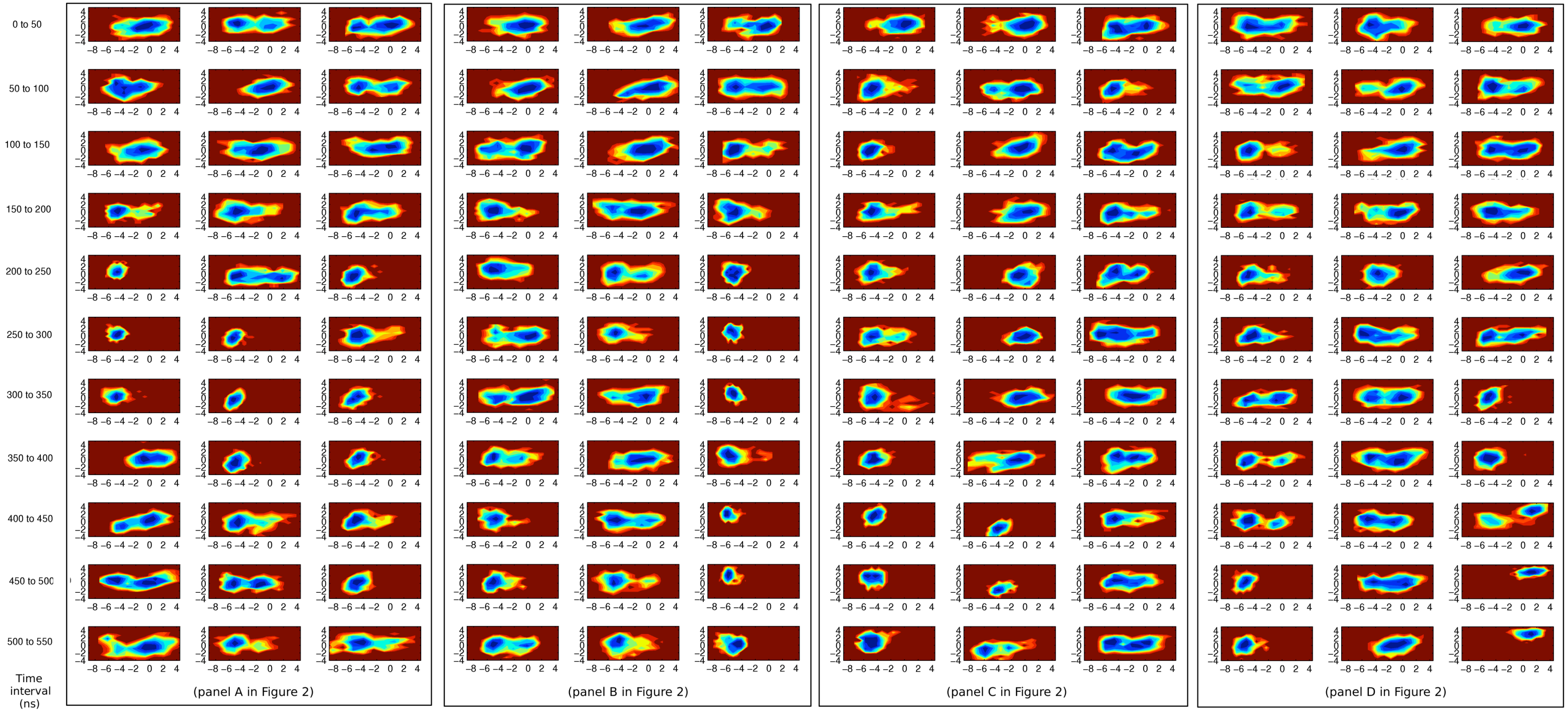

Figure S3. Internal dynamics of the ubiquitin molecules. Conformational space covered by the three protein molecules in 50 ns intervals of the four simulations presented in Figure 2, computed as projections on the first two principal components that describe ubiquitin variability across X-ray structures. After 200 ns, all molecules experience perturbations in their exploration of the conformational space due to their interactions with each other. In some cases a tight conformational restriction is observed, which is sometimes perturbed by internal reorientations or by binding of a third molecule.

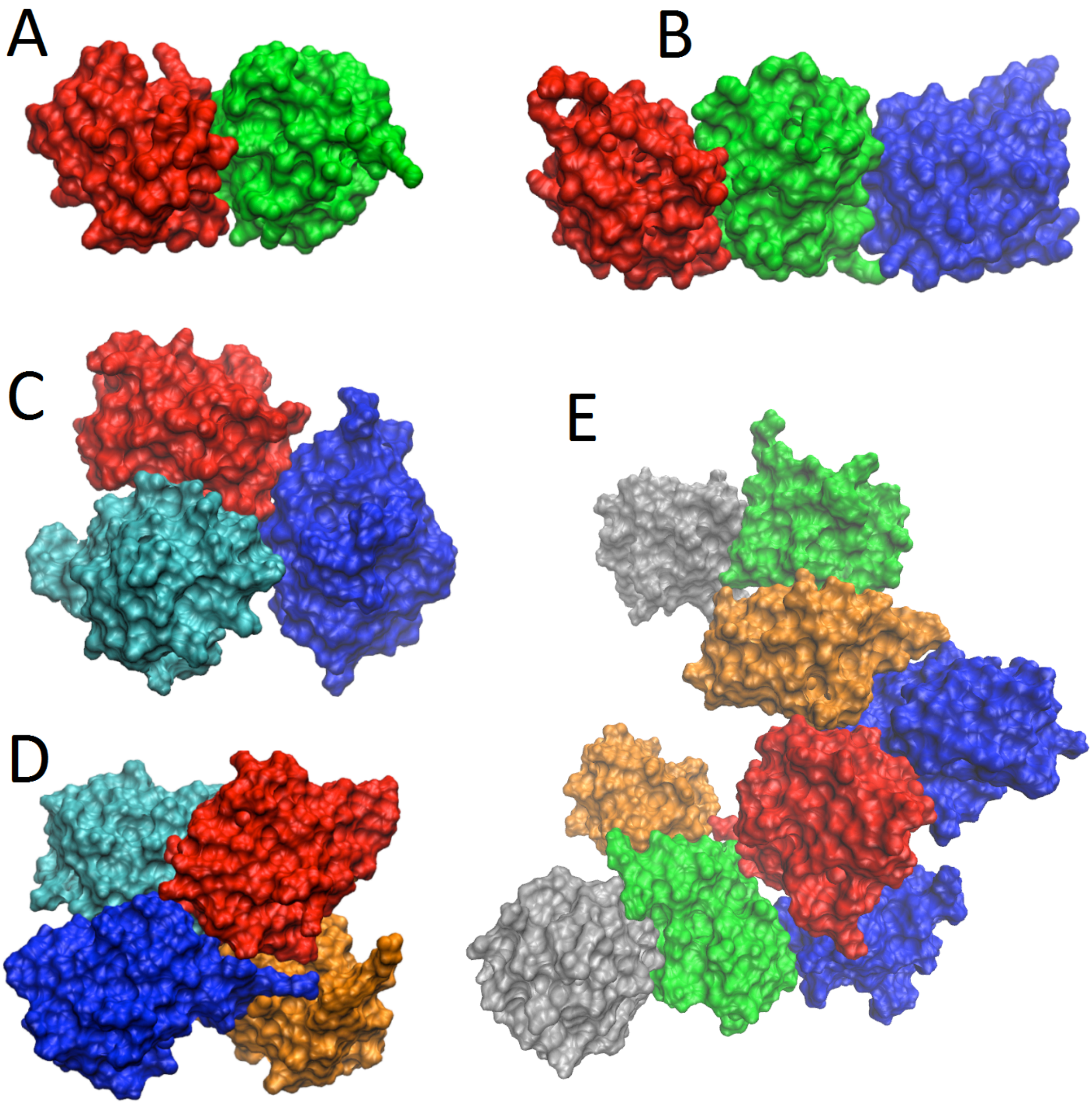

Figure S4. Different aggregates observed in free simulations. (A and B) examples of dimer and trimer formed in simulations with three protein molecules, (C and D) trimer and tetramer formed in the simulation with four ubiquitin molecules, (E) 9-mer formed in the simulation with 9 protein molecules.

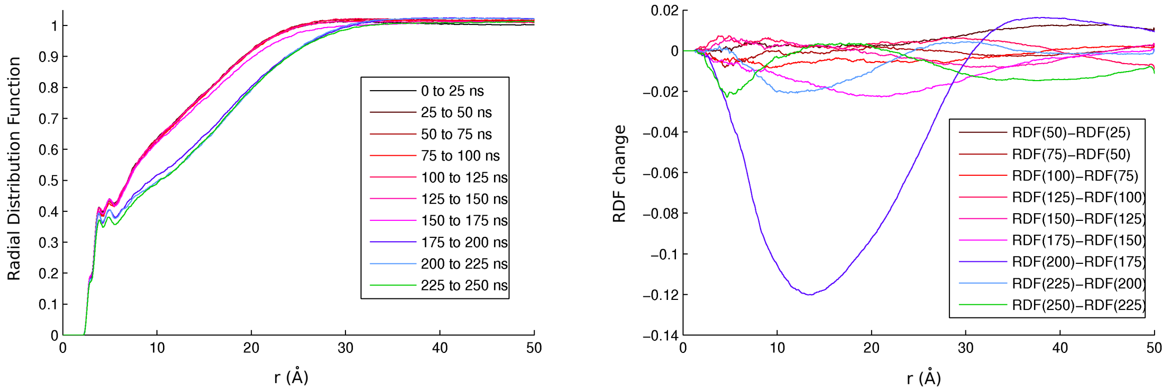

Figure S5. Water displacement upon binding of molecules 1 and 2 in the NMR-compatible event described in Figure 4. (A) Radial distribution function (RDF) of water molecules relative to residues of molecules 1 and 2 that are involved in persistent contacts in the noncovalent dimer, computed over 25 ns windows of the trajectory. (B) Differences between consecutive traces of panel A, to highlight changes in water structure happening throughout the trajectory. Roughly no perturbations of water structure occur up to 150 ns; by 150-175 ns water molecules located far from the surfaces start to move out of the way (pink traces) with a peak dehydration rate by 175-200 ns (blue); finally, water molecules from the first few hydration layers are also expelled (cyan and green).
